# Painful periods in adolescents

**DOI:** 10.1503/cmaj.201972

**Published:** 2021-04-19

**Authors:** Olga Kciuk, Sari Kives

**Affiliations:** Department of Obstetrics and Gynaecology, University of Toronto (Kciuk, Kives); The Hospital for Sick Children, Toronto (Kives), Toronto, Ont.

## Dysmenorrhea, defined as pain with menstruation, affects 50%–90% of menstruating adolescents and is the leading cause of recurrent school absenteeism in this population

One-third of menstruating adolescents miss school or sports because of dysmenorrhea.^1^ To minimize impact on academic and social development, physicians should proactively counsel patients on options for managing period pain.^1^–^3^ First-line treatment can be started before a specific diagnosis is made.^2^

## Up to 90% of dysmenorrhea in adolescents occurs in the absence of pelvic pathology (primary dysmenorrhea) and is mediated by excess prostaglandin production

Features suggestive of secondary dysmenorrhea (Box 1) should prompt pelvic ultrasonography and may warrant referral to a gynecologist.^2^,^3^

Box 1: **Features suggestive of secondary dysmenorrhea^2^,^3^**

- Onset immediately with menarche
- Progressively worsening dysmenorrhea
- Abnormal bleeding (including irregular bleeding) with pain
- Family or personal history of renal or other congenital anomalies (including spine, cardiac, or gastrointestinal)
- Midcycle or acyclic pain
- Dyspareunia
- Family history of endometriosis

## First-line treatment is short-term use of prophylactic nonsteroidal anti-inflammatory drugs (NSAIDs)

Naproxen, ibuprofen and other NSAIDs are equally effective, with a number needed to treat (NNT) of 3 to achieve pain relief in people with primary dysmenorrhea.^4^ Full-strength doses should be taken with food, on a regular schedule with no skipped administrations starting 1–2 days before the onset of menses (if predictable) or at the first sign of bleeding or pain, and continued for the first 2–3 days of bleeding.^2^,^3^

## Combined or progestin-only hormonal medications can be added as adjunct therapy

Combined oral contraceptives have an NNT of 5 for treating primary dysmenorrhea.^5^ Combined oral contraceptives with doses of ethinylestradiol above 30 μg should be chosen for adolescents, for maintenance of bone health.^6^ Continuous dosing provides more effective relief than standard cyclic use.^2^,^3^ Levonorgestrel-containing intrauterine systems and the etonogestrel implant are also safe and effective first-line options.^2^,^3^,^7^

## Endometriosis is the most common cause of secondary dysmenorrhea in adolescents

Endometriosis is found in as many as 70% of adolescents who undergo laparoscopy for dysmenorrhea refractory to treatment with NSAIDs and hormonal therapy.^3^ If dysmenorrhea persists beyond 3 months despite adequate first-line treatment, referral to a gynecologist is warranted.
